# The tongue biofilm metatranscriptome identifies metabolic pathways associated with the presence or absence of halitosis

**DOI:** 10.1038/s41522-022-00364-2

**Published:** 2022-12-19

**Authors:** M. Carda-Diéguez, B.T. Rosier, S. Lloret, C. Llena, A. Mira

**Affiliations:** 1 Genomics & Health Department, FISABIO Institute, Valencia, Spain; 2 Department of Stomatology, University of Valencia, Valencia, Spain; 3 CIBER of Epidemiology and Public Health, Madrid, Spain; 4 School of Health and Welfare, University of Jönköping, Jönköping, Sweden

**Keywords:** Clinical microbiology, Dental conditions, Infectious-disease diagnostics

## Abstract

Intra-oral halitosis usually results from the production of volatile sulfur compounds, such as methyl mercaptan and hydrogen sulfide, by the tongue microbiota. There are currently no reports on the microbial gene-expression profiles of the tongue microbiota in halitosis. In this study, we performed RNAseq of tongue coating samples from individuals with and without halitosis. The activity of *Streptococcus* (including *S. parasanguinis*)*, Veillonella* (including *V. dispar*) and *Rothia* (including *R. mucilaginosa*) was associated with halitosis-free individuals while *Prevotella* (including *P. shahi*), *Fusobacterium* (including *F. nucleatum*) and *Leptotrichia* were associated with halitosis. Interestingly, the metatranscriptome of patients that only had halitosis levels of methyl mercaptan was similar to that of halitosis-free individuals. Finally, gene expression profiles showed a significant over-expression of genes involved in L-cysteine and L-homocysteine synthesis, as well as nitrate reduction genes, in halitosis-free individuals and an over-expression of genes responsible for cysteine degradation into hydrogen sulfide in halitosis patients.

## Introduction

Halitosis is a highly prevalent condition characterized by oral malodor^1^. A Swedish study with more than 800 participants showed a 2% prevalence, while in a Chinese population with 2500 participants the estimated proportion increased up to 27.5%^2,3^. A recent systematic revision estimated a 31.8% prevalence of halitosis after examining 548 publications^4^. Depending on the origin of the oral malodor, halitosis can be differentiated into intra-oral (90% of cases), extra-oral and transient halitosis^5^. Several factors have been shown to affect bad breath, including necrotic pulpal exposure, deep carious lesions, specific food items, oral infections, periodontal disease, faulty restorations, reduced salivary flow, smoking and poor oral hygiene^6–10^. The oral malodor derives from the increase in the levels of several volatile sulfur compounds (VSCs), such as dimethyl sulfide (“DMS”, [CH_3_]_2_S), hydrogen sulfide (H_2_S) and methyl mercaptan (CH_3_SH). The last two have been unequivocally related to intra-oral halitosis and appear to be responsible for up to 90% of oral VSCs^11^ whereas dimethyl sulfide is considered to be implicated mainly in extra-oral blood borne halitosis^12^. Current clinical literature establishes specific threshold values for halitosis diagnosis for H_2_S (112 ppb), CH_3_SH (26 ppb) and [CH_3_]_2_S (8 ppb)^13–15^. Previous epidemiological reports suggest that other odoriferous molecules could also be responsible or contribute to bad breath, including volatile aromatic compounds and (poly)amines, short/medium-chain fatty acids or organic acids, alcohols, volatile aliphatic compounds, aldehydes and ketones^16^. Diverse microbiological and epidemiological studies have shown that VSCs are mainly produced by oral microbiota degradation of cysteine, cystine and methionine, as well as tryptophan, arginine and lysine (see Bollen et al. 2012 for a review)^17^. Most intra-oral halitosis is associated with microbial activity on the tongue and it has been reported that tongue coating in halitosis patients is thicker than that in healthy individuals^18,19^. Periodontal pockets can also be a source of halitosis and it is interesting that tongue coating in periodontitis patients was found to be 6 times more abundant compared to healthy individuals^20^, suggesting that both conditions could be interconnected. It has also been shown that hydrogen sulfide has cytotoxic and pro-inflammatory properties under certain conditions, potentially contributing to inflammation and tissue damage in periodontitis^21,22^, and therefore halitosis could have an effect on human health beyond the undesirable consequences of oral bad odor.

Microbial communities associated with caries, periodontitis and halitosis are known to have different compositions and functions in comparison to biofilms of healthy individuals, as a consequence of a microbial dysbiosis^23,24^. For example, species from *Neisseria* and *Rothia* are commonly found in higher concentration in healthy individuals in comparison to those with oral disease^25,26^. When the tongue microbiota of halitosis patients is compared to that of halitosis-free individuals, VSCs producers such as *Prevotella, Fusobacterium* and *Leptotrichia* are detected in higher proportions^5,27,28^.

The relationship of tongue- and saliva-associated microbiotas with halitosis has been studied recently using high-throughput sequencing approaches. In 2016, Ren and colleagues^5^ studied the tongue- and saliva-associated microbiotas in Chinese children with halitosis using 16S rRNA sequencing and shotgun metagenomics (direct, whole DNA sequencing) to study the changes in microbiota composition and functions. Similarly, Ye et al. (2019) used 16S rRNA sequencing in Chinese adults^29^. Seerangaiyan et al. used metabolomics to detect putative molecules associated with tongue coating and halitosis^30^. However, there is currently no evidence that tongue-associated microbes are over-expressing genes involved in VSC production in halitosis patients and it has not been determined if there are other activated functions or metabolic pathways that could favor the disease. The application of current RNAseq technologies to oral samples, by which the total metatranscriptomic pool from microbial communities is analyzed^31,32^, would allow the detection and quantification of all metabolic pathways that could be activated and repressed in halitosis patients.

In the current study, we have sequenced the total RNA of the microbial communities associated with tongue coating to provide the first metatranscriptomic profile of tongue-associated biofilms under halitosis-free and halitosis conditions. In addition, we identified three groups of halitosis patients with H_2_S, CH_3_SH or both above the established threshold, which were analyzed separately with the purpose of relating microbial activity to specific metabolic pathways, thereby shedding light to the underlying mechanisms of VSC production and to potential therapeutic approaches.

## Results

### Habits, oral conditions and VSCs

Several parameters have been associated with halitosis and VSC production, including tongue coating appearance, oral hygiene habits, oral pathologies and certain dietary components, such as dairy products, spicy foods, garlic and onion^17,33^. The effects of these parameters on the VSC levels in our study were evaluated (Table 1, Supplementary Table 1). However, no significant changes were found between the VSC levels of individuals in different groups based on any of these parameters (Table 1). For example, as expected, the average levels of VSCs (H_2_S and CH_3_SH) were higher in individuals with greater tongue coating extension (>1/3 of tongue) or thicker tongue coating compared to individuals with less tongue coating extension (<1/3 of tongue) or thin tongue coating, respectively, but these differences were not significant. Other possible confounding factors, such as systemic diseases, digestive problems, medication, use of spices, consumption of garlic, onion, alcohol or coffee, smoking, salivary flow, tooth brushing, tongue brushing, caries lesions, gingival inflammation or presence of a dry mouth, did not vary significantly between groups (Supplementary Table 1, *p* > 0.05 in all cases, t-test), suggesting that the VSCs profiles could primarily be assigned to changes in biofilm expression profiles.

**Table 1:** summary of clinical parameters in the healthy control group and halitosis groups.

Parameters			Control*^1^ (*n* = 16)	MM (*n* = 14)	HS (*n* = 10)	MM-HS (*n* = 10)	DMS (*n* = 33)	All (*n* = 83)
Gender	Male	*N* (%)	5 (31.3%)	3 (21.4%)	3 (30.0%)	5 (50.0%)	14 (42.4%)	30 (36.1%)
	Female	*N* (%)	11 (68.8%)	11 (78.6%)	7 (70.0%)	5 (50.0%)	19 (57.6%)	53 (63.9%)
Age (years)	Average (SD)		47.0 (13.9)	47.0 (13.6)	34.9 (13.2)	38.7 (16.4)	46.9 (12.7)	44.5 (14.0)
H2S (ppb)*^2^	Average (SD)		29.2 (32.6)	28.8 (26.1)	170.23 (36.4)	1237.38 (1428.4)	41.1 (31.3)	196.4 (614.0)
MM (ppb)	Average (SD)		7.1 (6.3)	72.5 (58.2)	14.38 (7.7)	280.52 (250.0)	5.5 (6.4)	51.3 (123.7)
DMS (ppb)	Average (SD)		4.7 (2.4)	23.2 (18.2)	67.01 (79.4)	79.75 (77.8)	47.6 (35.8)	41.4 (50.0)
Tongue colour	White	*N* (%)	8 (50.0%)	9 (64.3%)	7 (70.0%)	7 (70.0%)	18 (54.5%)	49 (59.0%)
	Light yellow	*N* (%)	3 (18.8%)	1 (7.1%)	0 (0.0%)	0 (0.0%)	4 (12.1%)	8 (9.6%)
	Dark yellow	*N* (%)	5 (31.3%)	4 (28.6%)	3 (30.0%)	3 (30.0%)	11 (33.3%)	26 (31.3%)
Tongue coating thickness	None	*N* (%)	0 (0.0%)	0 (0.0%)	0 (0.0%)	0 (0.0%)	2 (6.1%)	2 (2.4%)
	Thin	*N* (%)	9 (56.3%)	7 (50.0%)	8 (80.0%)	3 (30.0%)	14 (42.4%)	41 (49.4%)
	Moderate	*N* (%)	5 (31.3%)	7 (50.0%)	1 (10.0%)	7 (70.0%)	16 (48.5%)	36 (43.4%)
	Thick	*N* (%)	2 (12.5%)	0 (0.0%)	1 (10.0%)	0 (0.0%)	1 (3.0%)	4 (4.8%)
Tongue coating extension	None	*N* (%)	0 (0.0%)	0 (0.0%)	0 (0.0%)	0 (0.0%)	1 (3.0%)	1 (1.2%)
	<1/3 of tongue	*N* (%)	8 (50.0%)	6 (42.9%)	5 (50.0%)	2 (20.0%)	8 (24.2%)	29 (34.9%)
	1/3–2/3 of tongue	*N* (%)	7 (43.8%)	5 (35.7%)	4 (40.0%)	6 (60.0%)	20 (60.6%)	42 (50.6%)
	>2/3 of tongue	*N* (%)	1 (6.3%)	3 (21.4%)	1 (10.0%)	2 (20.0%)	4 (12.1%)	11 (13.3%)
Cavitated teeth*3	Yes	*N* (%)	2 (12.5%)	2 (14.3%)	3 (30.0%)	1 (10.0%)	3 (9.1%)	11 (13.3%)
	No	*N* (%)	14 (87.5%)	12 (85.7%)	7 (70.0%)	9 (90.0%)	30 (90.9%)	72 (86.7%)
Bleeding any site	Yes	*N* (%)	2 (12.5%)	3 (21.4%)	2 (20.0%)	3 (30.0%)	3 (9.1%)	13 (15.7%)
	No	*N* (%)	14 (87.5%)	11 (78.6%)	8 (80.0%)	7 (70.0%)	30 (90.9%)	70 (84.3%)
Periodontal pockets ≥4 mm any site	Yes	*N* (%)	4 (25.0%)	5 (35.7%)	2 (20.0%)	4 (40.0%)	8 (24.2%)	23 (27.7%)
	No	*N* (%)	12 (75.0%)	9 (64.3%)	8 (80.0%)	6 (60.0%)	25 (75.8%)	60 (72.3%)

*^1^In the first row, the groups are shown (Control is the halitosis-free group, MM is the methyl mercaptan group, HS is the hydrogen sulfide group, MM-HS is the group with both previous gasses above the halitosis threshold, and DMS is the dimethyl sulfide group).

*^2^H2S is hydrogen sulfide in parts per billion (ppb).

*^3^Cavitated teeth was considered any caries with ICDAS II score 5 or 6.

### Sequencing results

**Fig. 1:**
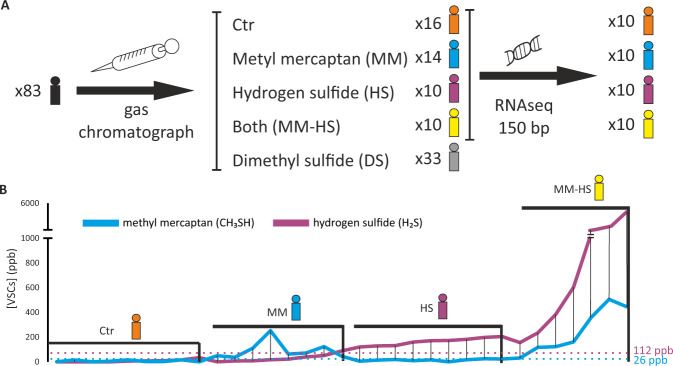
Study design for the metatranscriptomic analysis of tongue coating in halitosis. **A** The concentrations of three Volatile Sulfur Compounds (VSCs) in breath samples from 83 individuals was measured by gas chromatography and based on the levels of those gases, different groups were differentiated. Tongue biofilm samples were collected, and the extracted RNA was sequenced in 10 individuals per group. **B** The concentrations (ppb) of the two main intra-oral VSCs, methyl mercaptan and hydrogen sulfide, are shown for the 40 individuals whose transcriptomic profile was analyzed. The dotted lines indicate the consensus threshold concentration for halitosis.

Due to low RNA quality hindering library construction or to a low number of quality-filtered reads, the final number of samples per group was 9, 8, 9 and 7 for *Ctr*, *MM*, *HS* and *MM-HS* groups, respectively (Fig. 1A, B). The clinical parameters in each group are described in Table 1. After quality-filtering, an average of 3.7 × 10^3^ Gbp were obtained per sample. On average, 0.1% of reads belonged to the host and 93.5% of reads were lost after ribosomal RNA elimination. Once filtered, an average of 0.7 million mRNA reads per sample were annotated (53% of the filtered sequences) and used for the metatranscriptomic analysis of the tongue microbiota.

In order to study bacterial diversity and richness, we calculated the Shannon and Chao1 indexes based on the number of bacterial species (taxonomic diversity) and the number of annotated genes (functional diversity) (Supplementary Fig. 1). The rarefaction curves indicate that most diversity is detected by the sequencing effort performed. Interestingly, significant differences were only found at the genes level between *HS* and *MM-HS* groups (Shannon index) and between *Ctr* and *HS* groups (for the Chao1 index).

### Changes in active microbial proportions

**Fig. 2:**
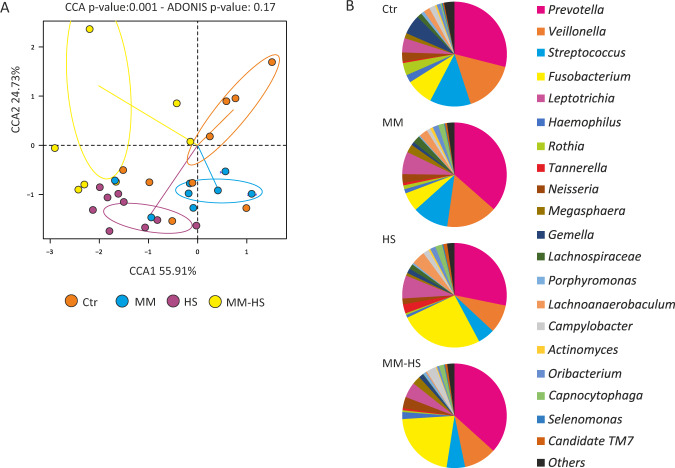
Profiles of active microbiota in healthy and halitosis groups. Active microbiota composition, as determined by the metatransciptomic data, was compared between groups in a CCA plot using ADONIS test **A**. The pie charts represent the top-20 most abundant genera in each group **B**. Ctr Control group, MM methyl mercaptan group, HS hydrogen sulfide group, MM-HS methyl mercaptan+hydrogen sulfide group (both gases over the halitosis threshold). The CCA *p*-values (ADONIS permanova) indicated that all groups differed significantly from each other (*p* < 0.007), except Ctr and MM (*p* = 0.078).

According to the Canonical Correspondence Analysis (CCA) a significant clustering between groups was obtained at the species taxonomic composition (*p* = 0.001) although a non-significant effect was detected by an ADONIS test (*p* = 0.17), probably due to a high intragroup variation within the *Ctr* and *MM*-*HS* groups (Fig. 2A). Inter-group differences were most apparent between the *Ctr* and the *HS* groups (*p* = 0.001) and the *Ctr* and the *MM-HS* groups (*p* = 0.001), which were both separated by the CCA1 component.

The proportion of each taxon was calculated to evaluate the RNA-based composition of the bacterial tongue communities. At the genus level, *Streptococcus* and *Rothia* were more active in *Ctr* while *Fusobacterium* was detected at higher levels in *HS* or *MM*-*HS* groups (Fig. 2B). However, some bacteria potentially associated to halitosis, such as *Fusobacterium* or *Prevotella*, are highly diverse, and the search for potential disease or health biomarkers should therefore be performed at lower taxonomic levels. At species level, an *unclassified Prevotella* sp.*, Streptococcus parasanguinis, Veillonella dispar, Veillonella atypica, Fusobacterium periodonticum, Prevotella histicola*, an unclassified *Veillonella sp., Streptoccocus salivarius, Rothia mucilaginosa, Prevotella melaninogenica* and an unclassified *Tannerella* sp. together accounted for 50–60% of all bacterial communities within all four groups (see Supplementary Table 2 for a full list).

**Fig. 3:**
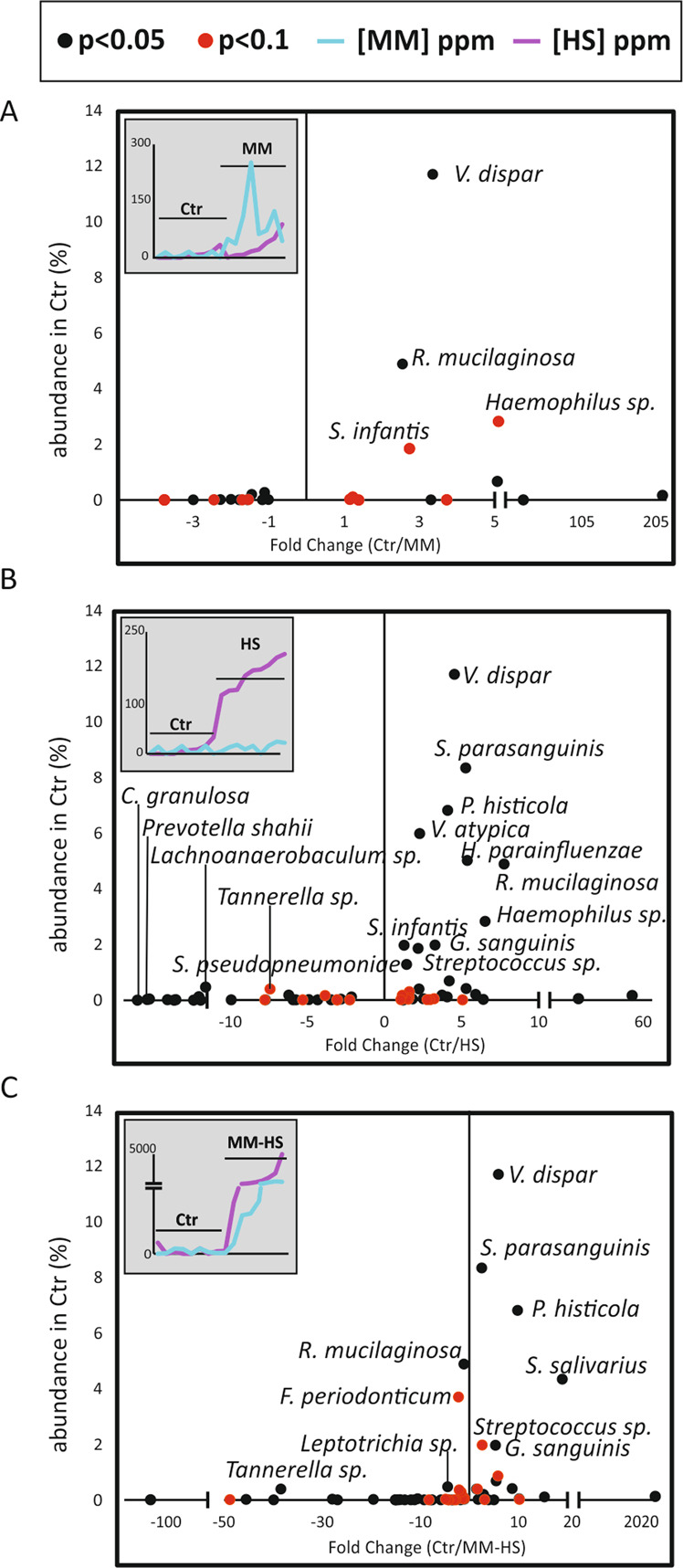
Microbial differences between control and halitosis metatranscriptomes. The graph shows bacterial species with significant differences (*p* < 0.05; DESeq2 test) or a statistical trend (0.05 < *p* < 0.1) between the control group and MM (**A**), HS (**B**) or MM-HS (**C**) groups. The Y-axis represents the abundance of each bacterial species in the control group, whereas the X-axis represents the difference in abundance as fold-change (Control/halitosis). The names of those species with >1% abundance are indicated. The concentrations of VSCs in the samples from the two groups compared are also shown, for reference.

When comparing the proportion of RNA-based microbial communities associated to the tongue of halitosis-free individuals and those with elevated VSCs concentrations, several significant differences were found (Fig. 3 and Supplementary Tables 3–5). As expected, the previously reported H_2_S producer *Fusobacterium periodonticum* was over-represented in the *MM-HS* group compared to the *Ctr* group (Fig. 3). Additionally, even though it was not significant, *F. periodonticum* showed a trend of higher representation (*p* = 0.1) in the *HS* group in comparison to the *Ctr* and *MM* groups. Additionally, other *Fusobacterium* species showed the same pattern as *F. periodonticum*, including *F. nucleatum* and an unclassified *Fusobacterium* sp. (Supplementary Tables 4–7**)**. Interestingly, these species were also significantly over-represented in the *MM-HS* group in comparison to the *MM* group and the *HS* group (Supplementary Tables 6 and 7).

**Fig. 4:**
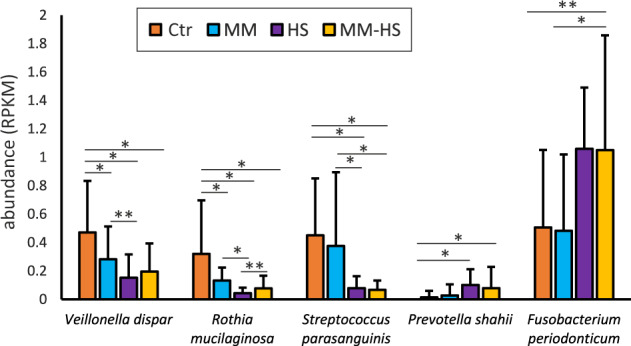
Halitosis and halitosis-free bacterial biomarkers. The bar graphs show bacteria with a significant difference in abundance in the metatranscriptome between control and halitosis patients, which could serve as potential biomarkers of health and halitosis in tongue biofilms. Three potential halitosis-free biomarkers (*V. dispar*, *R. mucilaginosa* and *S. parasanguinis*) and two potential halitosis biomarkers (*P. shahii* and *F. periodonticum*) are plotted for each identified group. RPKM: Number of mRNA reads normalized by gene length (Kbp) and by sequence coverage (Mbp). *p < 0.05 (DESeq2 test); **p < 0.1 (DESeq2 test). Standard deviation was used for error bars.

Similarly, several *Prevotella* species were significantly more active in the *MM-HS* group than in the *Ctr* group. They included *P. oulorum, P. sacharolytica, P. shahii* and *P. tannerae* (Supplementary Table 5). In addition, other *Prevotella* species were more active in the *HS* group than the *Ctr* group: *P. baroniae¸ P. denticola, P. shahii* and *P*. *tannerae* (Supplementary Table 4). These results suggest an association of active *Prevotella* and *Fusobacterium* species to tongue microbiotas with high concentration of H_2_S. Interestingly, we found *Prevotella histicola* associated with *Ctr* and *MM* patients in comparison to samples with high concentrations of H_2_S (Fig. 3, Supplementary Tables 4–5 and 6–8). In contrast to *Fusobacterium* and *Prevotella*, several *Streptococcus* species, *Rothia mucilaginosa* and *Veillonella dispar* were under-represented in patients with higher concentrations of H_2_S (Fig. 3). In fact, *R. mucilaginosa, Streptococcus paransanguinis* and *V. dispar* were over-represented in halitosis-free individuals in comparison to the three halitosis groups and their mean abundance in the halitosis-free group was remarkably high (4.9, 8.34 and 11.7%, respectively) (Fig. 4).

### H_2_S and amino acid biosynthesis in halitosis and halitosis-free patients

**Fig. 5:**
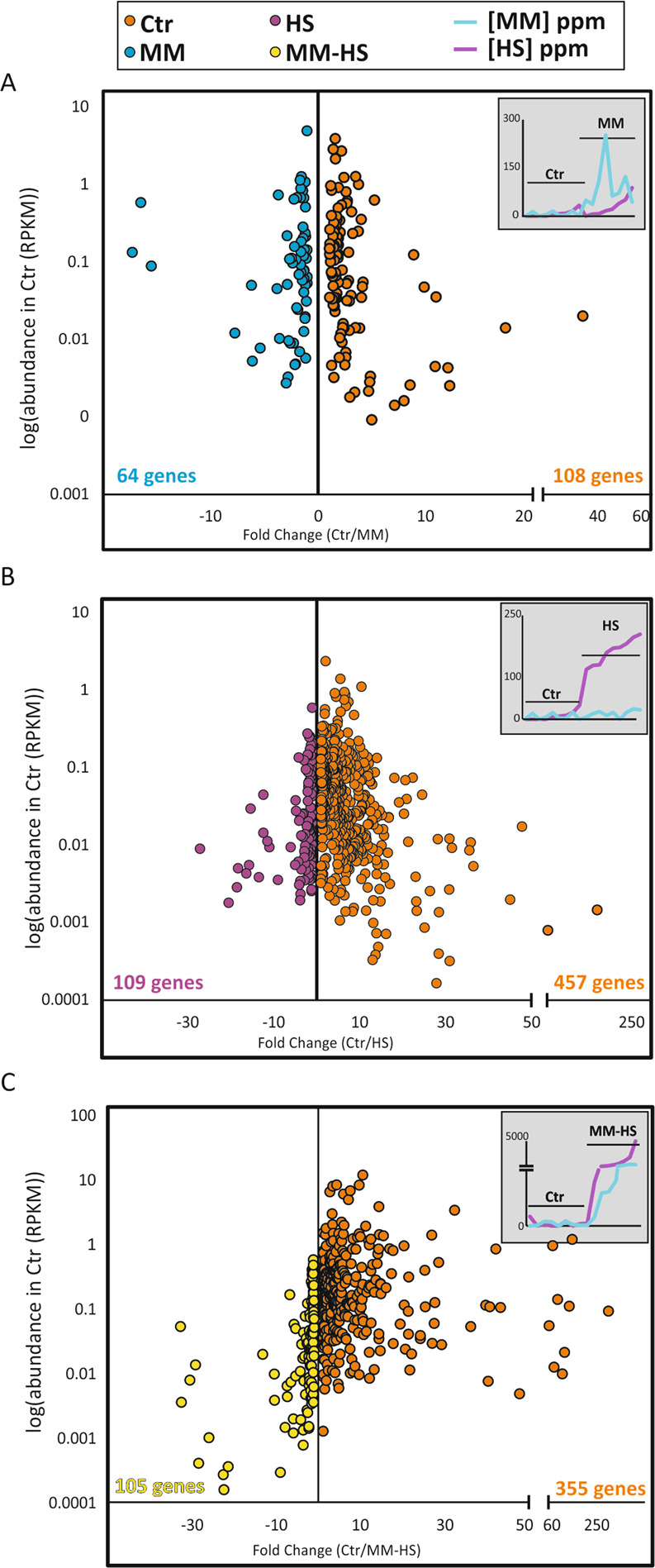
Microbial differences in gene expression (metatranscriptomic) profiles. The plots show genes with significant differences (*p* < 0.05; DESeq2 test) in expression between the control group and MM (**A**), HS (**B**) or MM-HS (**C**) groups. The Y-axis indicates the abundance of each gene in the control group whereas the X-axis represents the difference in gene expression, expressed as fold change (Control/Halitosis). The concentrations of VSCs in each sample from the two groups compared are also plotted, for reference. The total number of genes significantly over-expressed in each group is indicated for each comparison in blue and red. RPKM: Number of mRNA reads normalized by gene length (Kbp) and by sequence coverage (Mbp).

Besides the taxonomical composition we also studied the variation in gene expression. CCA plots show that at a functional level the microbiotas had different gene expression profiles depending on H_2_S concentration (Supplementary Fig. 2). When comparing different groups with DESeq2, several genes were significantly over- or under-represented (Supplementary Tables 9–14). In particular, the major differences were found when comparing either the *Ctr* or *MM* group to either the *HS* or *MM-HS* group (Fig. 5). Similar to what we observed using the taxonomical data, the differences between the *Ctr* group and the *MM* group (172 differentially represented genes, DRG) were smaller than those between the *Ctr* group and the *HS* group (566 DRG) or between the *Ctr* group and the *MM-HS* group (460 DRG).

**Fig. 6:**
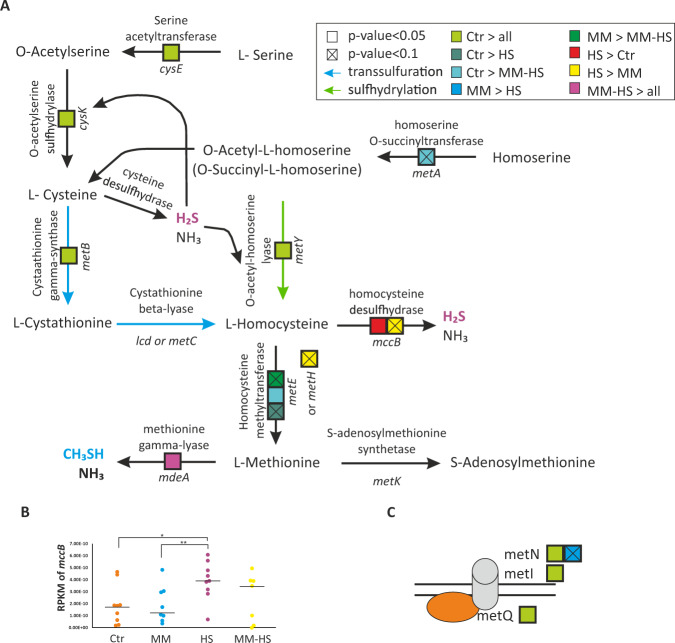
Gene expression profiles in the metabolic route involved in VSCs formation. The diagram represents the genes involved in the formation of HS and MM (**A**). The over-expression of a gene in a group is indicated with a coloured box. The expression levels (measured as RPKM) for the gene *mccB* are indicated for each group (**B**). Differences in the expression of the three subunits of the methionine membrane transporter are also shown (**C**). RPKM: Number of mRNA reads normalized by gene length (Kbp) and by sequence coverage (Mbp). DESeq2 test was used to calculate the *p*-values.

A major issue relates to whether genes coding for proteins responsible for H_2_S synthesis were over-expressed in the *HS* or *MM-HS* groups. According to previous reports, bacteria are able to produce H_2_S using two pathways: reduction of sulfate, and desulphydration of cysteine and methionine^34^, each of them presenting two possible routes. In the case of sulfate reduction, there are assimilatory and dissimilatory sulfate reduction pathways^35^. The desulphydration process includes a reverse trans-sulfuration and a cysteine transamination. Regarding the assimilatory pathway, we only found *cysC* (adenylyl-sulfate kinase) over-represented in the *HS* group compared to the *Ctr* group out of the 6 genes in this route (Fig. 6A). Interestingly, the homocysteine desulfhydrase (*mccB*), which is one of the three genes responsible for the L-homocysteine conversion into H_2_S, was over-represented in patients classified as *HS* (Fig. 6A and Supplementary Tables 10 and 12). There was also a 1.31 fold change difference in *mccB* expression between the *MM-HS* group and the *Ctr* group but the difference was not significant (Fig. 6B).

Even though the concentration of CH_3_SH was high enough to be considered as halitosis in *MM* and *MM-HS* groups, the mean concentration of CH_3_SH in the *MM-HS* group was 2.65 times higher than in the *MM* group (Fig. 1B, Table 1 and Supplementary Table 1). Among the genes coding for the enzymes responsible for CH_3_SH synthesis, we found *mdeA* (methionine gamma-lyase) over-expressed in the *MM-HS* group in comparison to the other three groups (Fig. 5A and Supplementary Tables 11, 13 and 14). According to this, the increase in CH_3_SH concentration could partly be due to the over-expression of this gene.

Among the genes over-represented in halitosis-free patients, we found the 3 subunits from the D-methionine transport system (*metQIN*) (Fig. 6C and Supplementary Tables 10 and 11). Interestingly, these 3 subunits were over-represented in halitosis-free individuals in comparison to both *HS* and *MM-HS* groups, suggesting an important role in oral health. Interestingly, *metY* (O-acetylhomoserine [thiol]-liase), which is responsible for the formation of L-homocysteine using H_2_S, was over-represented in *Ctr* samples when compared to *HS* and *MM-HS* groups (Fig. 6A and Supplementary Tables 10 and 11). Moreover, the utilization of sulfide as a sulfur source to form L-cystathionine was also favored in *Ctr* as indicated by the over-representation of *cysE* (Serine acetyltransferase), *cysK* (cysteine synthase) and *metB* (cystathionine gamma-synthase) in comparison to *HS* and *MM-HS* groups. A full statistical assessment of all genes found to be over- or under-expressed under each condition is shown in the Supplementary Dataset.

### Nitrate reduction is under-expressed in Halitosis patients

**Fig. 7:**
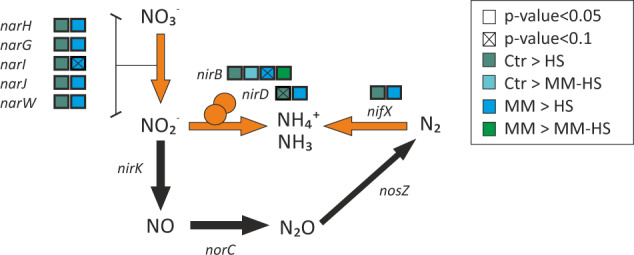
Nitrate metabolic pathways and halitosis. The diagram represents the nitrate metabolism reactions that were differentially expressed (orange arrows) between the tongue metatranscriptome of halitosis-free individuals and halitosis patients, as well as additional reactions that involved nitrogen-related metabolites (black arrows). The over-expression (DESeq2 test) of a gene in a group is indicated by a coloured box.

Genes involved in the reduction of nitrate (*narH, narG, narI, narJ and narW*) into nitrite were over-expressed in *Ctr* and *MM* groups in comparison to the *HS* group (Fig. 7). Apart from these, the genes coding for nitrogen (N_2_) fixation into ammonium (*nifX*) and the reduction of nitrite into ammonium (*nirB* and *nirD*) were also over-expressed in halitosis-free individuals in comparison to the *HS* group. In summary, reduction of nitrate into nitrite and the formation of ammonium appears to be favored in the oral cavity of halitosis-free individuals. This agrees with the taxonomic analysis described above where two nitrate reducing species, *Rothia mucilaginosa* and *Veillonella dispar*, and a third species that has also shown to produce nitrite (possibly by nitrate reduction), *Streptococcus parasanguinis*^36^, were more active in halitosis-free individuals.

### Classifying halitosis patients based on the VSC profile

This is the first study including a comparison in tongue microbial composition and activity between halitosis-free subjects and patients with high concentrations of specific VSCs, which included CH_3_SH only (*MM* group) but not H_2_S, or patients with high concentrations of H_2_S only (HS group). Surprisingly, 5 out of 9 *Ctr* individuals clustered within the *MM* group in the CCA plot at the species level (Fig. 2A), and in fact inter-group differences were not significantly different (*p* = 0.07). At a functional level, the differences between the *Ctr* and *MM* groups were significantly different (*p* = 0.01) (Supplementary Fig. 2). Moreover, the number of species (Fig. 3) and genes (Fig. 5) differentially represented between patients with high concentration of only CH_3_SH and the *Ctr* individuals was noticeably low in comparison to the *Ctr* vs the *HS* group or the *Ctr* vs the *MM-HS* group. Finally, CCA plots showed the *HS* group significantly separated from the *Ctr* and *MM* groups, especially at the gene level, suggesting that H_2_S but not CH_3_SH were associated with a significant change in the active microbiota.

## Discussion

In the present study, we have analyzed the composition and gene expression of the active tongue microbiota in halitosis-free individuals and halitosis patients using a metatranscriptomic approach. As this condition is the consequence of VSCs production, we measured the concentration of these gases and placed volunteers in four groups based on their VSC profiles. Independently of the group, the composition of the tongue microbiota detected in this study presented similarities to previously reported tongue communities by other authors^24,37–39^. Interestingly, a recent spatial-ecological study of the tongue-dorsum microbiota found a lower proportion of *Prevotella* and *Fusobacterium* than our work^40^. However, that study analyzed DNA instead of RNA, indicating that there these two bacterial genera could be proportionally more active than previously anticipated. RNA analysis is a powerful tool for assessing gene expression profiles but the results are circumscribed to the specific timepoint of the sampling; DNA analysis, on the other hand, shows gene presence and therefore functional potential, but lacks information about functional activity. Thus, using DNA or RNA depends on the hypothesis to test and metagenomic and metatranscriptomic approaches should be seen as complementary methodologies^41^

The analyses of differentially expressed genes suggested that halitosis was associated with communities that degrade amino acids and reduce sulfide, whereas tongue communities that produce L-cysteine from hydrogen sulfide and that reduce nitrate were associated with the absence of halitosis. This is the first time that the expression of these genes is shown to be related to the presence or absence of VSCs and halitosis. We, therefore, propose that this could provide new strategies to diagnose and/or treat this condition. It must be taken into consideration the fact that we did not measure the concentration of ammonium/ammonia in the tongue coating of those patients, and this compound has been found to be related to tongue coating and to the levels of VSCs in some studies^42^. We found that genes responsible for the conversion of nitrite into ammonium were more expressed in halitosis-free biofilms. That does not necessary imply that ammonia levels were higher in halitosis-free individuals since ammonia can be produced through other pathways such as amino acid degradation which is more common in proteolytic bacteria such as *Prevotella* or *Fusobacterium*. In addition, ammonium can also be assimilated for amino acid synthesis. We therefore propose that the gene that converts nitrite into ammonium is overrepresented because there is more substrate (nitrite) resulting from nitrate reduction but we do not know if ammonium/ammonia oral levels were higher in halitosis-free individuals.

An important drawback of the current study is the limited sample size. Given that there were no previous metatranscriptomic data, we could not perform power estimates to select an appropriate sample size and decided to perform an exploratory study with 10 individuals per group following power estimates from other metatranscriptomic data in dental plaque biofilms^32^. Now, based on the tongue biofilm metatranscriptomic data generated, we have performed a power analysis for multiple genes, which shows that the number of samples included in the analysis is below the one recommended by the power analysis. This could partly explain why some clinical parameters (e.g. tongue biofilm thickness) were, as expected, correlated with the levels of VSCs but the difference was not statistically significant. The limited sample size also implies that for the metatranscriptomic analysis, only genes with a clear and consistent association could be detected, underestimating the number of genes with over- or under-expression in the transcriptomic profile. Thus, future analysis with larger sample size and in different populations that could vary in dietary, cultural or genetic factors should be performed in order to confirm and expand the list of pathways and bacterial species associated to halitosis.

The comparison of active bacteria between halitosis-free individuals and those with high concentration of CH_3_SH and/or H_2_S supported previous studies in which known VSCs producers (e.g. *Porphyromonas, Fusobacterium* and *Prevotella* species) were associated with a high concentration of H_2_S^5,43–45^. Moreover, we detected an association of halitosis-free individuals with common oral commensals such as representatives of *Streptococcus*, *Corynebacterium*, *Rothia* and *Veillonella*. In agreement with this, Seerangaiyan et al. (2017) found an increase in several *Streptococcus* OTUs and a *Rothia dentocariosa* OTU in halitosis-free individuals compared to individuals with halitosis^46^. Similarly, the work of Zhang et al. (2021) has recently associated the abundance of *Rothia mucilaginosa* and *Streptococcus parasanguinis* with halitosis-free individuals^39^.

Interesting similarities were found between our results and those of Takeshita and colleagues (2012), who also classified patients in different groups based on their VSCs profiles, but used bacterial characterization by 16S rRNA pyrosequencing in saliva samples^15^. For example, *Porphyromonas and Fusobacterium* were associated in both studies with higher concentration of H_2_S. Similarly, *Veillonella* was found associated with the group with higher concentration of CH_3_SH in comparison to those with H_2_S. A difference was found when comparing the *MM* group with the *Ctr* group in our study, namely that *Veillonella* species were more active in halitosis-free individuals, but in their study more *Veillonella* was found in the MM group. Additionally, Takeshita et al. associated *Neisseria* with higher concentration of H_2_S. In our study, some species of *Neisseria* (with highest similarity to genes of *N. lactamica, N. mucosa, N. gonorrhoeae* and *N. meningitidis*) were more active in *HS* and *MM-HS* groups whereas others were more active in *MM* individuals compared to *HS* and *MM-HS* (*N. subflava, N. bacilliformis* and *N. polysaccharea*).

Interestingly, in our study, no differences in the estimated number of bacterial species were found between health and halitosis conditions. However, a statistically higher diversity in gene functions was found in *HS* individuals compared to healthy controls, suggesting that a larger set of metabolic pathways are activated under that halitosis condition.

One of the main findings in our analysis was the correlation of bacterial activity profiles with different VSC levels. According to the differences observed in active microbial proportions, the increase in H_2_S concentration was related to clear taxonomic changes associated to disease (i.e., dysbiosis), while this bacterial composition shift was modest when only CH_3_SH was elevated. In fact, patients with an increase in CH_3_SH but not H_2_S did not differ significantly in microbiota composition compared to halitosis-free individuals. We hypothesize that CH_3_SH in halitosis is a secondary metabolite of tongue-associated microbiota, but the presence of CH_3_SH by itself may not be an indication of dysbiosis. Thus, our data indicate that the MM group is similar to health in terms of bacterial activity and future research should determine if individuals with elevated levels of methyl mercaptan are perceived differently organoleptically with or without hydrogen sulfide above the halitosis threshold.

Previous epidemiological reports have observed a correlation of halitosis with periodontitis^2,18,47,48^. Both patients with halitosis and patients with gum disease have more tongue coating than halitosis-free individuals^5,47^. In our dataset, we did not find significant differences in tongue coating between halitosis-free and any of the 3 halitosis groups (*p* > 0.1). Remarkably, there are several periodontal pathogens among the mentioned species associated with malodor found in this study, such as representatives of *Porphyromonas*, *Fusobacterium* or *Prevotella*. These results in active microbial communities support the idea of an overlap in the bacterial composition of halitosis and periodontitis, and a possible link between these diseases^18,47^.

The periodontitis-associated genera that were higher in the halitosis groups (*Porphyromonas*, *Fusobacterium* and *Prevotella*) included species which are known to produce VSCs (i.e. *Porph. gingivalis, F. nucleatum*, and *Prev. intermedia*)^28,49^ and have been commonly detected in higher amounts in the tongue of halitosis patients^5,24,46^. Thus, we propose these species as potential universal halitosis biomarkers that increase in this condition. On the other hand, we detected *Veillonella dispar, Streptococcus parasanguinis* and *Rothia mucilaginosa* significantly more active in individuals classified as halitosis-free. These are commensal members of the oral microbiota and *Streptococcus* and *Rothia* have previously been detected in higher proportions in groups with lower VSCs concentration and organoleptic scores^39,50^. Interestingly, *Veillonella* species have been found in dental cavities and have been reported to produce H_2_S in vitro^51^, but have recently also been found more abundant in the tongue microbiota of halitosis-free individuals^48^. In our study, *V. dispar* was more active in individuals that did not produce significant amounts of H_2_S. A possible explanation is that in halitosis-free in vivo conditions, *V. dispar* does not produce H_2_S, while in disease conditions, other species (e.g., *Fusobacterium* spp. and *Prevotella* spp.) use cysteine and/or reduce sulfate more efficiently. It could also be possible that *V. dispar* is producing VSCs but at a concentration below the established thresholds or that in the presence of nitrate, sulfate reduction is inhibited by more energy-efficient nitrate reduction^52^. The higher activity of *R. mucilaginosa*, which is a highly efficient nitrate reducer^53^, in halitosis-free individuals, also suggests that nitrate could favor health-associated conditions. Taken together, our data suggest that nitrate could have a beneficial effect on tongue communities involved in halitosis, as previously suggested^26,54^. This agrees with the well-known phenomenon that reducing nitrate provides more energy than reducing sulfate, and therefore microorganisms normally switch to denitrification when nitrate is available, which could therefore imply lower VSC production^52^. Secondly, nitrite is reduced to nitric oxide by several oral bacteria, and nitrite reducing species can use hydrogen sulfide as electron donor, which would therefore lower the levels of H_2_S in the oral environment^26,55^. Another indirect suggestion comes from sewage treatment plants, where nitrate can be used as a biological agent to treat malodor by limiting microbial VSCs production that results from sulfate reduction^52^. Therefore, we propose *V. dispar, S. parasanguinis* and *R. mucilaginosa* as biomarkers for halitosis-free individuals and suggest nitrate as a potential prebiotic to promote a halitosis-free microbiome. Because of the limitation at the statistical power of this study, future experimental and clinical work should test this hypothesis both experimentally and in intervention studies.

In conclusion, this is the first report of microbial gene expression profiles in the tongue biofilm of halitosis patients. The comparison of these with halitosis-free individuals indicated that an elevated concentration of H_2_S (hydrogen sulfide), or its combination with CH_3_SH (methyl mercaptan), were associated with dysbiosis of the tongue microbiota. The changes in active microbiota composition associated with an increase in VSCs concentration are in accordance with previous reports based on DNA analysis. These include an increase in *Prevotella, Fusobacterium* and *Porphyromonas* and a reduction in commensal oral members such as *Streptococcus, Veillonella* and *Rothia*. Our metatranscriptomic data provide the first evidence that genes involved in VSCs production are being expressed in vivo and identify several routes by which the bacterial communities in halitosis-free individuals consume VSCs to transform them in non-halitosis compounds. Finally, we have detected a relationship between halitosis and a repression of genes responsible for nitrate reduction. Thus, we propose those pathways involved in VSC consumption and in nitrate reduction as potential therapeutic targets for preventing and treating the disease, which should be evaluated in future studies.

## Methods

### Study population

This study was performed at the University of Valencia Dental Clinic, managed by the Lluís Alcanyís Foundation (Valencia, Spain), and conducted according to the ethical principles of the Declaration of Helsinki of 2008. The study protocol was approved by the FISABIO-DGSP Ethical Committee (B1O2015-68711-R) and all participants signed an informed consent prior to sample donation. Potentially suitable individuals who visited the dental clinic for revision or treatment were invited to a screening for participation. The inclusion criteria for participants were between 18 and 65 years of age, systemically healthy, and presence of at least 20 teeth. Individuals were excluded if they suffered from any systemic disease (including liver or renal diseases, bronchial disorders, uncontrolled diabetes, gastroesophageal disease and reflux, cancer, autoimmune diseases, and anemia). Other exclusion criteria included pregnant or nursing women, as well as antibiotic treatment or regular antiseptic use in the last month. Participants who fulfilled the selection criteria received the following dietary instructions for the day of their visit, including avoidance of food with garlic, onion or strong spices, no alcohol consumption 24 h before the visit, no coffee consumption in the 6 h before the visit, no food or tobacco consumption 2 h before the visit, no teeth or tongue brushing or use of interdental cleaning elements on the day of the visit, and avoidance of products with mint or perfume.

### VSCs measurements and patient classification

The concentrations of three VSCs (H_2_S, CH_3_SH and [CH_3_]_2_S) in the breath of 83 individuals were measured (in ppb) using an Oralchroma portable gas chromatograph (Fig. 1 and Supplementary Table 1). For the VSCs measurement, patients were asked to breath in a sterile syringe that was immediately injected into the OralChroma and measurements were taken according to the manufacturer’s instructions. Based on the measurements of the two main intraoral halitosis gases, H_2_S and CH_3_SH, individuals were classified into four groups (Fig. 1): a control group (Ctr) of halitosis-free individuals with both gasses below the halitosis-threshold (<112 ppb for H_2_S and <26 ppb for CH_3_SH,^13–15^) and three halitosis groups, namely the MM group (<112 ppb H_2_S and >26 ppb CH_3_SH), the H_2_S group (>112 ppb H_2_S and <26 ppb CH_3_SH), and the MM-HS group (>112 ppb H_2_S and >26 ppb CH_3_SH). In each group, the 10 individuals that best fulfilled the criteria (i.e., lowest or highest ppb of one or both of the gasses) were selected for RNAseq analysis.

### Tongue microbiota sampling

Tongue coating samples (containing the tongue microbiota) were collected from the selected 40 participants from the middle and posterior sections of the tongue by scrapping with an autoclaved Heidemann spatula four times. Middle and posterior tongue samples from each individual were pooled. The samples were put into 0.5 ml of RNAlater stabilizing solution (Invitrogen, Thermo Fisher Scientific) and directly frozen at −20 °C until RNA extraction.

### Oral health assessment

A Periodontogram was used and a gingival index was determined following Löe et al. (1963) and Camelo-Castillo et al., (2015)^56,57^. Periodontal status was evaluated using the Community Periodontal Index (CPI)^58^. Presence of gingival bleeding and periodontal pockets were recorded. A metallic probe with a 0.5 mm ball tip was used, with a black band between 3.5 and 5.5 mm, and rings at 8.5 and 11.5 mm from the ball tip. Presence of cavitated caries lesion (ICDAS II codes 5 or 6), were assessed^59^. Unstimulated whole saliva was collected by spitting into graduated disposable plastic cups for 5 min. The flow rate was expressed in milliliters per minute^60^. The color of the tongue coating (white, light yellow, dark yellow, brown), its extension (one, two or three thirds) and its width (0: absence of tongue biofilm; 1: thin layer that allows visualization of papillae; 2: moderate layer, papillae are partially seen; 3: thick layer, papillae are not seen) were recorded^61^. This tongue coating evaluation was performed by the same dental professional, as some of these features, especially tongue color, may be subjective. In addition, the individuals filled out a questionnaire with information about oral hygiene and dietary questions. For this study, the analyzed parameters included tongue brushing (yes/no and frequency), dental flossing (yes/no) and mouthwash usage (yes/no). Additionally, the analyzed dietary parameters included protein-rich diet (yes/no) and the consumption of garlic or onion (garlic/onion/both), the consumption of spicy foods (yes/no) and dairy products (yes/no). The presence of removable dental prosthesis or orthodontics and the presence of xerostomia was also registered. A full description of clinical and dietary parameters is shown in Supplementary Table 1.

### Sampling, RNA extraction and sequencing

The tongue coating samples were thawed on ice and nucleic acids were manually extracted using the Master Pure Complete DNA and RNA Purification Kit (Epicentre), following the manufacturer protocol, and resuspended in a final volume of 30 µl. Every step during RNA extraction was performed with RNase-free, low-retention Eppendorf tubes and RNase-free water (Sigma). The DNA + RNA extracted was treated 3 times with a Turbo DNA-free Kit (Ambion) at 37 °C for 30 min each in order to eliminate DNA and keep RNA. DNA-free material was amplified (PCR) using universal 16S rRNA primers to confirm the absence of DNA traces^32^. Genomic DNA was used as positive control. The PCR program included 2 minutes at 94 °C, 30 cycles (94 °C 15”, 55 °C 30” and 72 °C 40”) and a final amplification step 72 °C for 5 min. The absence of a DNA band was considered evidence of correct DNAse treatment. RNA concentration was measured with Qubit RNA BR Assay Kit (ref:Q10210). Libraries and cDNA sequencing were performed at the FISABIO sequencing platform (Valencia, Spain) using NextSeq Illumina Technology (single pairs, High-input 1 × 150 bp) following the manufacturer’s instructions.

### Bioinformatic analysis

Reads were trimmed by quality and length using the PRINSEQ program^62^. Human and microbial ribosomal sequences were identified and separated by aligning the sequence dataset to the human genome (Bioproject PRJNA31257) and SILVA database, respectively^63^. Remaining reads were considered to be mRNA reads and were used for annotation. We used a manually curated version of the Human Oral Microbial Database (HOMD)^64^ to annotate the mRNA reads. The abundance of active bacteria was studied using the number of reads aligned to bacterial ORFs from the HOMD. To eliminate the potential bias caused by differences in gene length and dataset size, the number of reads annotated to a gene were normalized by its length (Kbp) and the size (in Megabp) of the dataset. Thus, the level of gene expression was indicated as number of reads per gene Kbp per Mbp (RPKM).

We assigned a KEGG model to every Open Reading Frame (ORF) by aligning the amino acid dataset to the KEGG database^65^ with HMMER^66^. The abundance of a fragment was considered to be equal to the number of sequences aligned into this fragment using BLAST^67^. The calculations for obtaining the abundance matrices and the comparative analyses (see below) were performed using custom R scripts (http://www.R-project.org/). Principal component and canonical correspondence correlation analyses were performed in order to display differences between the groups. DESeq2 test was used to calculate the significance of differences between groups^68^. Analyses were performed with the significance level of *p* < 0.05 (significant *p*-value) or *p* < 0.1 (statistical trend).

### Reporting summary

Further information on research design is available in the Nature Research Reporting Summary linked to this article.

## Supplementary information

Supplementary Material figures

Supplementary material tables

preprint in medrxiv.org

Reporting Summary

## Data Availability

The datasets generated during the current study are available in the SRA repository with the accession number PRJNA735705.
