# Supplementary material for: The tongue biofilm metatranscriptome identifies metabolic pathways associated with the presence or absence of halitosis
Source: NPJ Biofilms Microbiomes. 2022 Dec 19;8:100. doi: 10.1038/s41522-022-00364-2 (PMC9763428; doi:10.1038/s41522-022-00364-2)
Supplement: Supplementary file 1 — Supplementary Material figures [file 41522_2022_364_MOESM1_ESM.pdf]

Supplementary Figure 1. Bacterial and functional diversity in tongue biofilms from halitosis and halitosis-free individuals. Diversity (Shannon) and richness (Chao1) indexes were calculated using both the number of bacterial species (A) and genes (B) detected in the metatranscriptomes, obtained by Nextseq Illumina RNAseq. Rarefaction curves (C) were calculated for the same number of reads in each sample ($5x10^4$), annotated to a bacterial species (left) or a gene (right). Differences were calculated using Wilcoxon test.

Supplementary Figure 2. Functional differences between halitosis groups. The graph shows the results of a Canonical Correspondence Analysis (CCA) based on microbial gene expression in the different groups studied (*Ctr, MM, HS* and *MM-HS*). An ADONIS test was used.

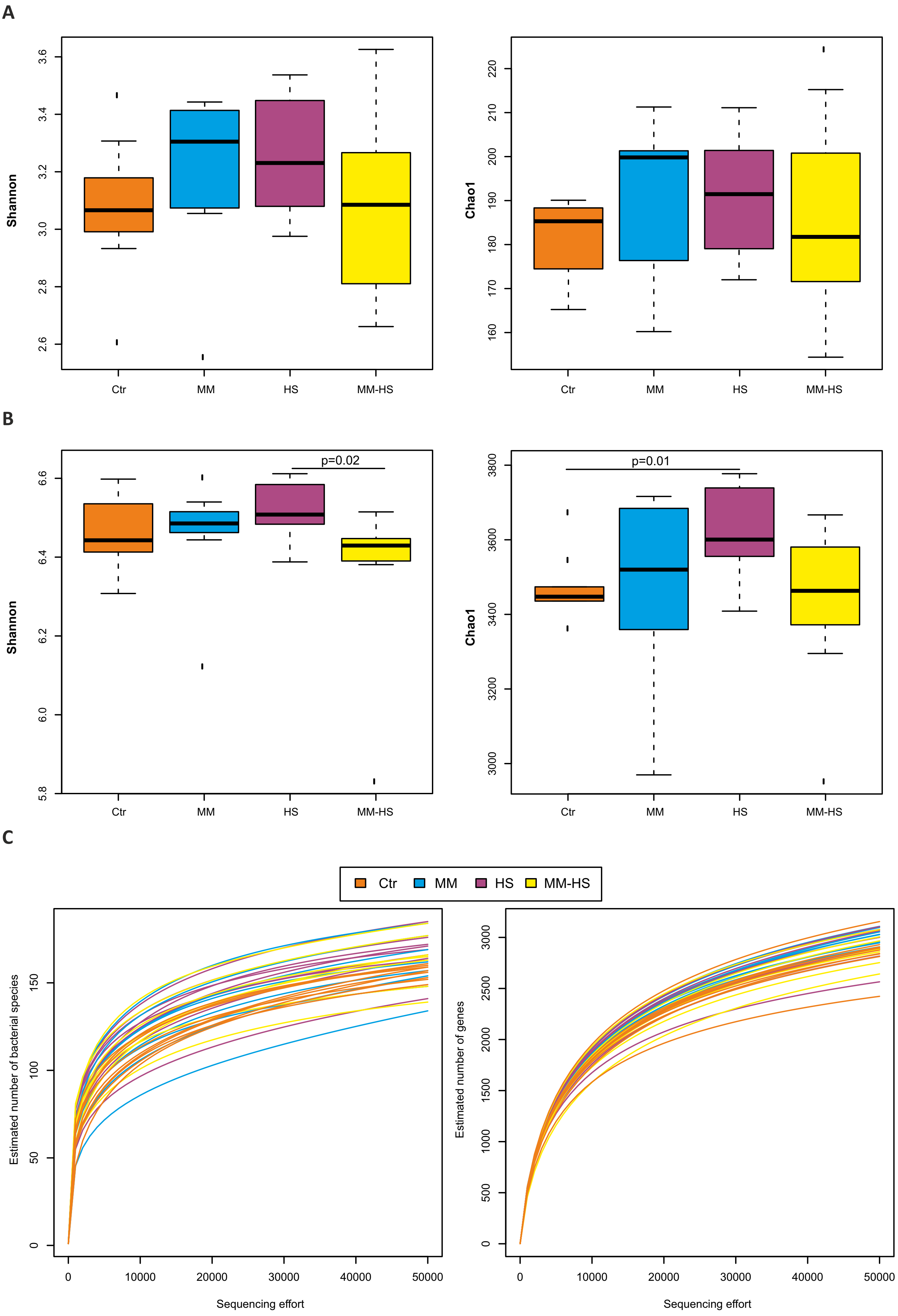
A
Shannon
3.6
3.4
3.2
3.0
2.8
2.6
Ctr
MM
HS
MM-HS
Chao1
220
210
200
190
180
170
160
B
Shannon
6.6
6.4
6.2
6.0
5.8
p=0.02
Chao1
3800
3600
3400
3200
3000
p=0.01
C
Ctr
MM
HS
MM-HS
Estimated number of bacterial species
0
50
100
150
0
10000
20000
30000
40000
50000
Sequencing effort
Estimated number of genes
0
500
1000
1500
2000
2500
3000
Sequencing effort

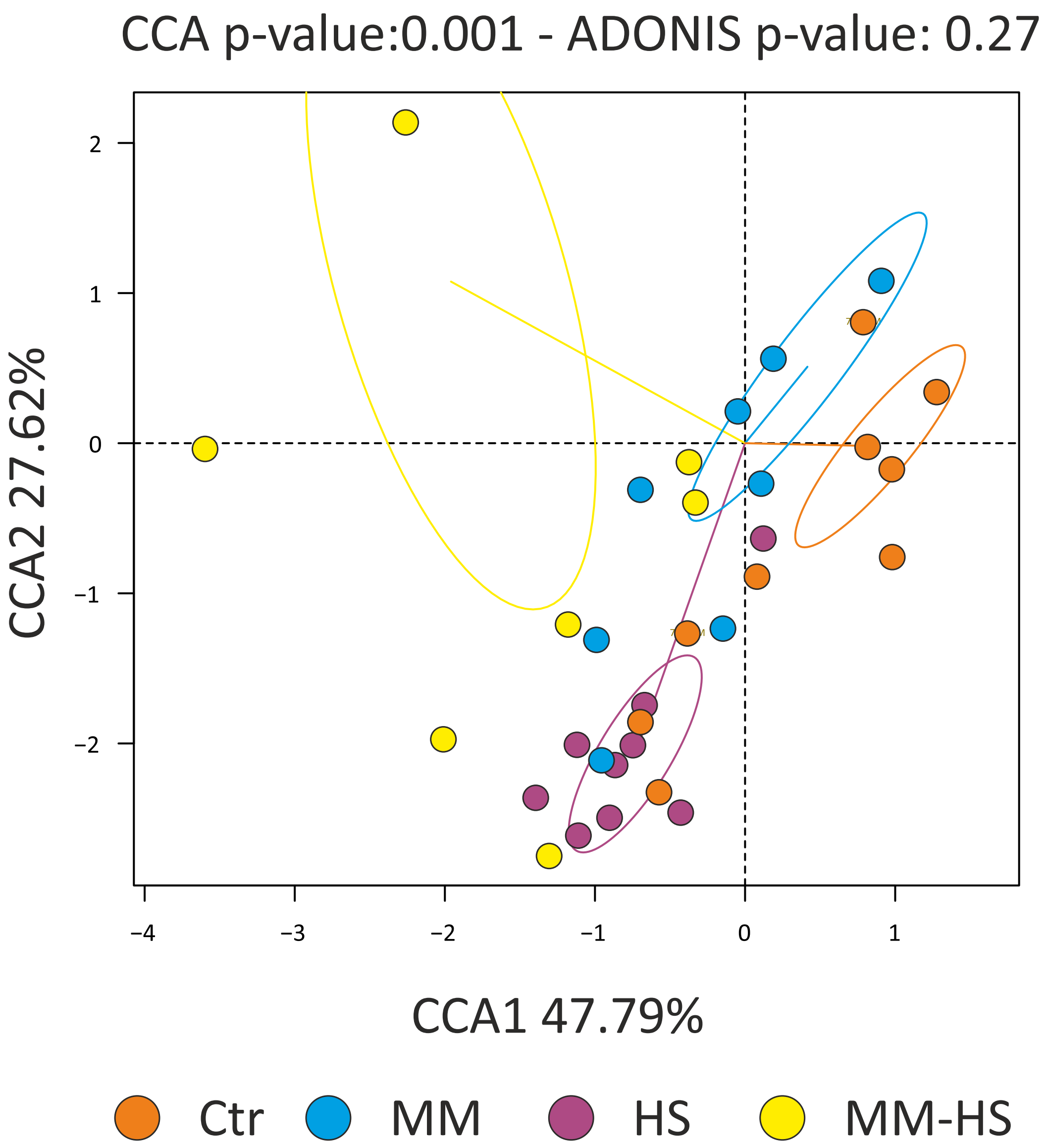
CCA p-value:0.001 - ADONIS p-value: 0.27
CCA2 27.62%
CCA1 47.79%
2
1
0
−1
−2
−4
−3
−2
−1
0
1
Ctr
MM
HS
MM-HS
