# Supplementary material for: The tongue biofilm metatranscriptome identifies metabolic pathways associated with the presence or absence of halitosis
Source: NPJ Biofilms Microbiomes. 2022 Dec 19;8:100. doi: 10.1038/s41522-022-00364-2 (PMC9763428; doi:10.1038/s41522-022-00364-2)
Supplement: Supplementary file 4 — Reporting Summary [file 41522_2022_364_MOESM4_ESM.pdf]

nature portfolio

ALEJANDRO|MIRA|OBRADOR

Firmado digitalmente por ALEJANDRO|MIRA|OBRADOR
Fecha: 2022.05.18 22:47:00 +02'00'

Corresponding author(s): Alex Mira Obrador

Last updated by author(s): May 11, 2022

Reporting Summary

Nature Portfolio wishes to improve the reproducibility of the work that we publish. This form provides structure for consistency and transparency in reporting. For further information on Nature Portfolio policies, see our Editorial Policies and the Editorial Policy Checklist.

Statistics

For all statistical analyses, confirm that the following items are present in the figure legend, table legend, main text, or Methods section.

n/a	Confirmed	
☐	☒	The exact sample size (*n*) for each experimental group/condition, given as a discrete number and unit of measurement
☐	☒	A statement on whether measurements were taken from distinct samples or whether the same sample was measured repeatedly
☐	☒	The statistical test(s) used AND whether they are one- or two-sided<br>*Only common tests should be described solely by name; describe more complex techniques in the Methods section.*
☐	☒	A description of all covariates tested
☐	☒	A description of any assumptions or corrections, such as tests of normality and adjustment for multiple comparisons
☐	☒	A full description of the statistical parameters including central tendency (e.g. means) or other basic estimates (e.g. regression coefficient) AND variation (e.g. standard deviation) or associated estimates of uncertainty (e.g. confidence intervals)
☐	☒	For null hypothesis testing, the test statistic (e.g. *F*, *t*, *r*) with confidence intervals, effect sizes, degrees of freedom and *P* value noted<br>*Give P values as exact values whenever suitable.*
☒	☐	For Bayesian analysis, information on the choice of priors and Markov chain Monte Carlo settings
☒	☐	For hierarchical and complex designs, identification of the appropriate level for tests and full reporting of outcomes
☐	☒	Estimates of effect sizes (e.g. Cohen's *d*, Pearson's *r*), indicating how they were calculated

Our web collection on statistics for biologists contains articles on many of the points above.

Software and code

Policy information about availability of computer code

Data collection	*Provide a description of all commercial, open source and custom code used to collect the data in this study, specifying the version used OR state that no software was used.*
Data analysis	*Provide a description of all commercial, open source and custom code used to analyse the data in this study, specifying the version used OR state that no software was used.*

For manuscripts utilizing custom algorithms or software that are central to the research but not yet described in published literature, software must be made available to editors and reviewers. We strongly encourage code deposition in a community repository (e.g. GitHub). See the Nature Portfolio guidelines for submitting code & software for further information.

Data

Policy information about availability of data

All manuscripts must include a data availability statement. This statement should provide the following information, where applicable:

- Accession codes, unique identifiers, or web links for publicly available datasets
- A description of any restrictions on data availability
- For clinical datasets or third party data, please ensure that the statement adheres to our policy

The datasets generated during the current study are available in the SRA repository with the accession number PRJNA735705.

Field-specific reporting

Please select the one below that is the best fit for your research. If you are not sure, read the appropriate sections before making your selection.

☒ Life sciences ☐ Behavioural & social sciences ☐ Ecological, evolutionary & environmental sciences

For a reference copy of the document with all sections, see nature.com/documents/nr-reporting-summary-flat.pdf

Life sciences study design

All studies must disclose on these points even when the disclosure is negative.

Sample size	Given that there were no previous metatranscriptomic data in tongue samples, we could not perform power estimates and decided to do an exploratory study with 10 individuals per group following power estimates from other metatranscriptomic data in dental plaque biofilms (Benitez-Paez et al. 2014).<br>Now that we have obtained the tongue biofilm metatranscriptomic data, we have performed a power analysis for those genes that were included in figure 6 and 7. The analyses estimated that 10 halitosis patients would be enough to reach this power in the HS and MM-HS groups but an N of 30 individuals would be required in halitosis-free individuals to reach a statistical power of 70%, due to higher variance in this group.
Data exclusions	Individuals were excluded if they suffered from any systemic disease (including liver or renal diseases, bronchial disorders, uncontrolled diabetes, gastroesophageal disease and reflux, cancer, autoimmune diseases, and anemia). Other exclusion criteria included pregnant or nursing women, as well as antibiotic treatment or regular antiseptic use in the last month.<br><br>In addition, samples with low sequencing coverage after host genome filtering and ribosomal RNA filtering were also removed.
Replication	VSCs measurments with Oralchroma were done by triplicate.
Randomization	This is not relevant. Participants were included in each of the 4 groups depending on the VSCs concentrations on their breaths.
Blinding	Blinding not applicable in this cross-sectional study because participants were grouped according to clinical parameters (not a clinical trial).

Reporting for specific materials, systems and methods

We require information from authors about some types of materials, experimental systems and methods used in many studies. Here, indicate whether each material, system or method listed is relevant to your study. If you are not sure if a list item applies to your research, read the appropriate section before selecting a response.

Materials & experimental systems

n/a	Involved in the study	
☒	☐	Antibodies
☒	☐	Eukaryotic cell lines
☒	☐	Palaeontology and archaeology
☒	☐	Animals and other organisms
☐	☒	Human research participants
☒	☐	Clinical data
☒	☐	Dual use research of concern

Methods

n/a	Involved in the study	
☒	☐	ChIP-seq
☒	☐	Flow cytometry
☒	☐	MRI-based neuroimaging

Human research participants

Policy information about studies involving human research participants

Population characteristics	The inclusion criteria for participants were between 18 and 65 years of age, systemically healthy, and presence of at least 20 teeth.
Recruitment	Potentially suitable individuals who visited the University of Valencia Dental Clinic for revision or treatment were invited to a screening for participation.
Ethics oversight	The study protocol was approved by the FISABIO-DGSP Ethical Committee (B1O2015-68711-R) and all participants signed an informed consent prior to sample donation.

Note that full information on the approval of the study protocol must also be provided in the manuscript.
